# Antimicrobial stewardship in community pharmacies in Uyo, Akwa Ibom State: practices, barriers, and facilitators

**DOI:** 10.1017/ash.2026.10773

**Published:** 2026-07-13

**Authors:** Mbuotidem Ime Akpan, Idongesit Linus Jackson, Unyime Israel Eshiet, Mary Richard Akpan

**Affiliations:** Department of Clinical Pharmacy and Biopharmacy, Faculty of Pharmacy, University of Uyo, Uyo, Akwa Ibom State, Nigeria

## Abstract

**Objective:**

To evaluate the practices, barriers, and facilitators related to antimicrobial stewardship (AMS) in community pharmacies (CPs).

**Design:**

Cross-sectional study

**Setting:**

CPs in Uyo, Akwa Ibom State, Nigeria

**Participants:**

One hundred and twelve pharmacists working in CPs, who had been practicing for at least six months and gave informed consent to participate, were surveyed.

**Methods:**

Potential participants were approached at their CPs during working hours and the study’s objectives explained to them. Data were collected using a 31-item self-administered questionnaire from October to November 2023 and analyzed with SPSS version 25.0.

**Result:**

Participants were predominantly male (61.6%). Eighty-seven (77.7%) indicated that they often/always encourage patients to run a culture/sensitivity test before taking antibiotics. Most (95.5%) indicated that their choice of antibiotics is based on such results. The most frequently cited barriers to implementing AMS in CPs were limited access to patient records (93.8%), unrestricted patient access to antimicrobials without a prescription (90.1%), and physicians’ resistance to pharmacists intervening in antibiotic selection (58.0%) or the dose/duration of prescribed antibiotics (50.9%). The most commonly reported facilitators of AMS were increased availability of pharmacy education programs on AMS (92.9%), improved access to patients’ clinical/laboratory data (88.4%), improved collaboration with physicians in hospitals (85.7%), and public awareness initiatives emphasizing the role of community pharmacists in AMS (85.7%).

**Conclusion:**

Participants reported generally favorable AMS practices. However, tailored AMS education programs for pharmacists and the general public, along with stronger collaboration between pharmacists and physicians and between CPs and hospitals are recommended.

## Introduction

Antimicrobial resistance (AMR) is a growing global health concern that contributed to approximately 4.95 million deaths worldwide in 2019 ^1^ and was associated with over 3 million deaths among children worldwide in 2022.^2^ If nothing is done, these figures could rise to 10 million annually by 2050, with devastating health and economic consequences.^3^ Two research studies conducted in the United States revealed that about 30%–50% of antibiotic prescriptions are inappropriate, highlighting the widespread reach of this challenge.^4,5^ Africa has also borne its share of this global menace, with an estimated 1.05 million deaths linked to AMR.^6^

The World Health Organisation (WHO) lists AMR as one of the top ten global public health threats facing humanity.^7^ In response, the WHO Global Action Plan on AMR adopted strategies to help curb these threats. The objectives of this Global Action Plan are to improve awareness and understanding of AMR through communication and training; strengthen knowledge via surveillance and research; reduce infection incidence through hygiene, sanitation, and infection prevention; optimize antimicrobial use through stewardship programs across human and animal health; and promote sustainable investment in new medicines, diagnostics, and vaccines.^8^ Collectively, these objectives support a coordinated, “One Health” approach to tackling AMR.^9^

Antimicrobial stewardship (AMS) refers to a coherent set of actions aimed at ensuring the appropriate use of antimicrobials to achieve safe and effective clinical outcomes.^10^ This is achieved through appropriate selection of antimicrobials, usually following a sensitivity test, as well as the correct dosing, route of administration, and duration of therapy, to achieve safe and effective clinical outcomes at a reasonable cost.^11^ Household-and community-level self-medication with antimicrobials remains a significant global driver of inappropriate use.^12^ A recent systematic review and meta-analysis revealed a pooled prevalence of self-medication at 43.0%, with the highest rate, 55.2%, observed in sub-Saharan Africa.^13^ Another systematic review spanning 24 countries reported a 62% pooled prevalence of non-prescription antibiotic dispensing for self-limiting conditions.^14^ The widespread dispensing of antibiotics without prescription at community pharmacies may enhance the spread of AMR, underscoring the need for targeted AMS initiatives at the community level.^14^

Pharmacists, especially in community settings, are easily accessible, frequently consulted, and trusted healthcare professionals known for resolving minor health conditions.^15^ Consequently, community pharmacists could play a crucial role in the success of initiatives aimed at combating AMR particularly in developing countries such as Nigeria, where the use of antibiotics without a prescription is predominant.^16^ Nonetheless, there are limited data regarding the practices, facilitators, and barriers related to AMS in community pharmacies in Nigeria. This study aimed to evaluate the practices, barriers, and facilitators related to AMS in community pharmacies in Uyo, Akwa Ibom State, Nigeria.

## Methods

### Design, setting, and participants

This was a descriptive cross-sectional study among 112 pharmacists across 95 community pharmacies in Uyo Local Government Area (LGA), Akwa Ibom State, Nigeria. Uyo, the state capital, covers approximately 214.31 km^2^ and has a population of about 1.5 mmillion.^17^ The minimum sample size of 86 was computed with an online sample size calculator,^18^ considering a 95% *c*onfidence level, a 5% margin of error, 110 registered community pharmacies in Uyo as of August 2023, and a 50% response distribution. To increase the statistical power of the analysis, however, 112 pharmacists drawn from 95 community pharmacies were surveyed. Pharmacists who had been practicing for at least six months and provided informed consent to participate were included in the study.

### Instrument for data collection

Data were collected using a 31-item self-administered questionnaire adapted from previous studies.^19–23^ To assess content validity, the 31-item instrument was sent to three academic pharmacists in the Department of Clinical Pharmacy and Biopharmacy, University of Uyo, Nigeria, who have experience in psychometrics and community practice. They were required to give their opinions with regard to the relevance and clarity of each item. Their feedback was used to modify and improve the instrument. The revised questionnaire was piloted among ten community pharmacists who were not included in the main study. These participants were asked to read through and complete the instrument and to state which item(s), if any, were ambiguous. Based on their feedback, final refinements were made before field administration. The questionnaire comprised two sections: Section A enquired about personal information of the participant, while Section B sought information regarding knowledge/awareness, perceptions, practices, barriers, and facilitators related to AMS in community pharmacies.

### Data collection

Potential participants were approached during working hours where the study’s objectives and procedures where explained. Eligible participants were required to fill the informed consent form for participation by ticking a box on the data collection instrument. The questionnaire was administered on-site and retrieved upon completion. The data collection period lasted from October to November 2023.

### Data analysis

Data were entered into Microsoft Excel and exported to SPSS version 25.0 (IBM Corp., Armonk, NY, USA) for analysis. Descriptive statistics were used to summarize the data. To simplify analysis and make results easier to interpret, the 5-point Likert response scales were reduced to 3-point scales. ‘Strongly agree’ and ‘agree’ were combined into ‘agree‘, while ‘strongly disagree‘ and ‘disagree‘ were merged into ‘disagree‘. The neutral option remained unchanged. In a similar manner, the original options ‘never‘ and ‘rarely‘ were combined into a single category (labeled never/rarely), while ‘often‘ and ‘always‘ were grouped as often/always; the ‘occasionally’ option was retained as a distinct option.

### Ethical considerations

Ethical approval was obtained from the Health Research Ethics Committee of the Ministry of Health, Akwa Ibom State. Informed consent was obtained from each participant.

## Results

Out of 125 community pharmacists approached, 112 agreed to participate, yielding a response rate of 89.6%. Thirteen pharmacists declined participation, citing time constraints or lack of interest.

### Characteristics of participants

Most of the participants were male (61.6%); only 8 (7.1%) of the participants were over 49 years old. Most of the participants (88.4%) had a Bachelor of Pharmacy (B.Pharm) or Doctor of Pharmacy (PharmD) as their highest qualification; 49 (43.8%) had less than 5 years’ experience in community practice. The majority of participants had heard of AMS (68.8%) and had read a document about it (65.2%) (Table 1).

**Table 1.** Characteristics of participants (N = 112)

Characteristic	Frequency	Percent
**Gender**		
Male	69	61.6
Female	41	36.6
**Age (years)**		
<30	40	35.7
30–39	47	42.0
40–49	17	15.2
≥50	8	7.1
**Highest Qualification**		
B. Pharm/PharmD	99	88.4
M. Pharm/M.Sc	8	7.1
FPCPharm	1	0.9
PhD	1	0.9
**Position**		
Locum Pharmacist	41	36.6
Superintendent pharmacist	42	37.5
Owner of premises	8	7.1
Owner of premises and superintendent pharmacist	21	18.8
**Length of experience (years)**		
<5	49	43.8
5–9	42	37.5
10–15	16	14.3
>15	10	8.9
**Ever heard about AMS**		
Yes	77	68.8
No	35	31.3
**Ever read a document on AMS**		
Yes	73	65.2
No	39	34.8
**Attended a training on AMS**		
Yes	40	35.7
No	72	64.3

*AMS* Antimicrobial stewardship, *B.Pharm* Bachelor of Pharmacy, *PharmD* Doctor of Pharmacy, *M.Pharm* Master of Pharmacy, *M.Sc* Master of Science, *FPCPharm* Fellow of the West African Postgraduate College of Pharmacists.

### Perceptions about antimicrobial resistance and antimicrobial stewardship

Most of the participants (86.6%) disagreed that antibiotic resistance is a problem in the hospital setting but not in community pharmacies. Only 4 (3.6%) disagreed that lack of microbiological testing contributes to AMR. The majority of participants (94.6%) agreed that community pharmacists can play an important role in AMS (Table 2).

**Table 2.** Perceptions of antimicrobial resistance and antimicrobial stewardship

Variable	Agree	Neutral	Disagree
Antibiotic resistance is a problem in the hospital setting but not in community pharmacies	10 (8.9)	5 (4.5)	97 (86.6)
Dispensing antibiotics without prescription from community pharmacies contributes to the problem of antibiotic resistance in Nigeria	79 (70.5)	11 (9.8)	22 (19.6)
Lack of microbiological testing contributes to antimicrobial resistance	106 (94.6)	2 (1.8)	4 (3.6)
AMS will reduce inappropriate antibiotic use	100 (89.3)	6 (5.4)	6 (5.4)
AMS will reduce health care costs associated with infections*	97 (87.4)	9 (8.1)	5 (4.5)
Continuous medical education would help achieve the goal of AMS*	103 (92.8)	6 (5.4)	2 (1.8)
Community pharmacists can play an important role in AMS	106 (94.6)	5 (4.5)	1 (0.9)
AMS will enhance pharmacists’ job satisfaction*	101 (91.0)	8 (7.2)	2 (1.8)

*AMS* Antimicrobial stewardship; *n = 111 due to non-response; values are n (%).

### Practices of participants

One hundred and two (91.1%) of the participants indicated that they often/always educate patients on the use of antimicrobials and resistance-related issues; about a third (33.9%) of them reported that they occasionally ask the patients about their knowledge of prescribed antimicrobials and their usage. Ninety-six (86.5%) agreed that they supply antibiotics only if they are certain that a patient has a bacterial infection. About half of the participants (54.4%) agreed that they would supply a broad-spectrum antibiotic when uncertain about the most appropriate antibiotic for a patient (Table 3).

**Table 3.** Practices of participants (N = 112)

Variable	Never/rarely	Occasionally	Often/always
I educate patients on the use of antimicrobials and resistance-related issues	2 (1.8)	8 (7.1)	102 (91.1)
I communicate with the prescriber whenever possible if I am unsure about the appropriateness of an antibiotic prescribed for a patient	13 (11.6)	35 (31.3)	64 (57.1)
I refer to treatment guidelines for infectious diseases when making a decision for antimicrobial use for my patient	3 (2.7)	23 (20.5)	86 (76.8)
I encourage patients to run a sensitivity test before taking antibiotics	2 (1.8)	23 (20.5)	87 (77.7)
I ask the patients about their knowledge of the prescribed antimicrobials and their usage	22 (19.6)	38 (33.9)	52 (46.4)

	Agree	Neutral	Disagree
If I am unsure whether or not a patient has a bacterial infection, I will supply antibiotics just in case it is	10 (8.9)	18 (16.1)	84 (75.0)
If I am in doubt which antibiotic is best for a patient, I will supply a broad-spectrum one, just in case	61 (54.4)	20 (17.9)	31 (27.7)
I supply antibiotics when patients request them specifically, because if I do not supply them, they will just go to another pharmacy	22 (19.6)	12 (10.7)	78 (69.6)
I supply antibiotics only if I am certain that a patient has a bacterial infection*	96 (86.5)	8 (7.2)	7 (6.3)

* n = 111 due to non-response; values are n (%).

**Figure 1.**
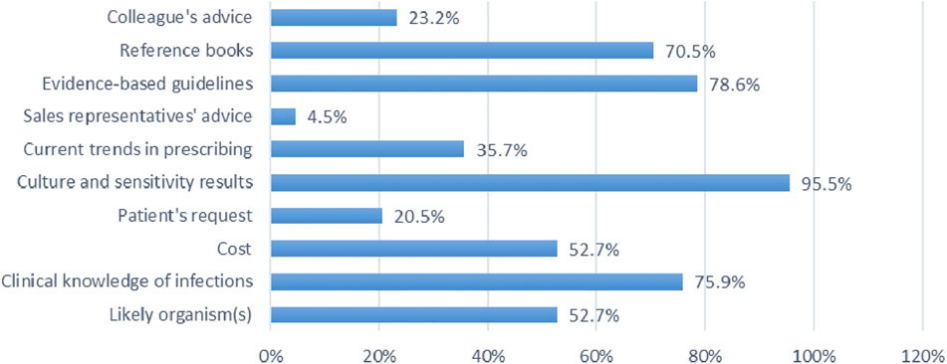
Factors influencing community pharmacists’ choice of antibiotics for patients.

A clear majority of the participants (95.5%) indicated that their choice of antibiotics for patients is based on microscopy, culture, and sensitivity tests, while only a few (4.5%) reported that advice from pharmaceutical/sales representatives influences their antibiotic choices (Figure 1).

### Barriers to antimicrobial stewardship in community pharmacies

The most frequently reported barriers to AMS in community pharmacies were limited access to patient records to evaluate the appropriateness of antibiotic prescriptions (93.8%), unrestricted patient access to antimicrobials without a prescription (90.1%), and a lack of receptiveness from physicians toward pharmacists intervening in the selection of antibiotics for patients (58.0%) (Table 4).

**Table 4.** Barriers to antimicrobial stewardship in community pharmacies

Variable	Agree	Neutral	Disagree
I do not have the required training to participate in AMS*	16 (14.4)	19 (17.1)	76 (68.5)
I do not have enough time to participate in AMS**	16 (14.5)	24 (21.8)	70 (63.6)
There are not any standard guidelines to implement AMS*	18 (16.2)	29 (26.1)	64 (57.7)
Patient access to antimicrobials without a prescription is a challenge to AMS in community pharmacies*	100 (90.1)	5 (4.5)	6 (5.4)
Limited access to patient records to evaluate the appropriateness of prescribed antibiotics is a challenge to AMS in community pharmacies	105 (93.8)	3 (2.7)	4 (3.6)
Physicians are not receptive to pharmacists intervening on the choice of antibiotics	65 (58.0)	24 (21.4)	23 (20.5)
Physicians are not receptive to pharmacists intervening on the dose and/or duration of antibiotics	57 (50.9)	28 (25.0)	27 (24.1)

*AMS* Antimicrobial stewardship; *n = 111 due to non-response; **n = 110 due to non-response; values are n (%).

### Facilitators of antimicrobial stewardship in community pharmacies

**Figure 2.**
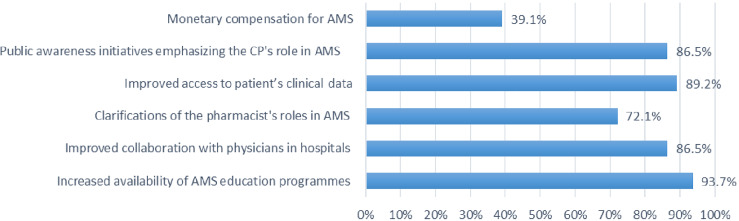
Facilitators of antimicrobial stewardship in community pharmacies AMS antimicrobial stewardship; CP community pharmacist.

The two most frequently cited facilitators of AMS in community pharmacies were increased availability of pharmacy education programs on AMS (93.7%) and better access to patient’s clinical and laboratory data (89.2%) (Figure 2).

## Discussion

This study explored the practices, barriers, and facilitators associated with AMS in community pharmacies in Uyo, Akwa Ibom State, Nigeria. Additionally, it assessed the participants’ knowledge and perceptions regarding AMS.

The participants reported generally favorable practices related to AMS. Most pharmacists reported that they would not dispense antibiotics “just in case” nor accede to patient-specific demands for antimicrobials. However, about half of them admitted to dispensing broad-spectrum antibiotics in cases of diagnostic uncertainty. This practice of dispensing broad-spectrum antibiotics when diagnosis is uncertain supports earlier report of widespread use of fluoroquinolones and penicillins among residents of the city where the current study was conducted.^24^ According to the authors, most antibiotics consumed were from the Watch category of the WHO AWaRe (Access, Watch, Reserve) classification, which has a higher resistance potential than the Access group.^24,25^ Similarly, a nationwide study of antibiotic dispensing among community pharmacists revealed that cephalosporins, penicillins, and quinolones were the major classes of antibiotics dispensed.^26^ Although most penicillins belong to the Access group, most cephalosporins and all quinolones generally fall under the Watch group.^25^ There is a need for antibiotic stewardship interventions aimed at educating community pharmacists about the WHO AWaRe classification of antibiotics and the importance of minimizing the dispensing of Watch-group antibiotics without a prescription or clear clinical indication.^27,28^ Targeted training has been reported to significantly improve dispensing appropriateness and reduce inappropriate antibiotic recommendations among community pharmacists.^28^

Most of the participants in our study reported that they regularly educate patients on proper antimicrobial use and resistance-related issues. This contrasts with reports from Pakistan^29^ where community pharmacists seldom provided such counseling. Strengthening patient education with formal support and resources would amplify its impact, as reported in previous research.^30^

The results of this study also indicate that most of the community pharmacists surveyed would encourage patients to run a sensitivity test before taking an antibiotic. This report supports findings from a previous study conducted in Western Nigeria, suggesting that community pharmacists in Nigeria understand the importance of a personalized treatment approach.^31^

In our study, limited access to patient records for evaluating the appropriateness of prescribed antibiotics was the most commonly cited barrier to implementing AMS in community pharmacies, consistent with a previous study that also identified this as a barrier.^32^ Most of our participants also reported that patient access to antimicrobials without a prescription poses a barrier to implementing AMS in community pharmacies. Unrestricted access to antimicrobials continues to pose a global barrier to attaining AMS goals, especially in low-and middle-income countries (LMICs).^27^ This highlights the need for stricter enforcement on the rational and clinical use of antimicrobials in attaining these goals. Another major barrier identified was physicians’ resistance to pharmacists intervening in antibiotic selection or the dose/duration of prescribed antibiotics. This echoes findings from an Australian study which revealed that pharmacists and physicians rarely collaborated on concerns related to AMS.^33^ It is crucial to develop and implement ongoing AMS education programs for healthcare professionals, including physicians, because AMS requires a multidisciplinary team effort across healthcare settings.

In spite of the importance of AMS education progrmmes, our study revealed that most of our participants had never attended a training on AMS. This gap likely reflects the limited integration of comprehensive AMS lectures in undergraduate pharmacy curricula in Nigeria.^34^ This observation also mirrors findings from Australia, where pharmacists reported insufficient training to confidently engage in AMS programs.^35^

Most pharmacists acknowledged AMR as a pressing issue in community pharmacies and recognized how a lack of microbiological diagnostics and non-prescription dispensing exacerbate AMR. Similar findings were reported by participants in a previous study.^36^ The overall high awareness of participants on AMS aligns with findings from Pakistan, where pharmacists exhibited good general knowledge of AMS despite misconceptions regarding resistance drivers.^21^

Key facilitators of AMS in community pharmacies identified in our study were enhanced availability of pharmacy education programs on AMS, improved access to patients’ clinical data, public awareness initiatives that emphasize the role of community pharmacists in AMS, and stronger collaboration with physicians in hospitals. Globally, pharmacists in LMICs show willingness to adopt AMS practices when supported with training, diagnostic tools, and interprofessional collaboration.^16,32^ Interestingly, only a small minority felt that monetary compensation was a motivating factor, a mindset equally shared by community pharmacists in a survey done in Thailand.^37^ Most participants also reported that AMS in community pharmacies will enhance pharmacists’ job satisfaction. These findings suggest that while financial recognition may be beneficial, pharmacists’ commitment appears to be largely driven by professional responsibility and a sense of service. These findings also reflect a widespread professional eagerness to engage in AMS, consistent with a previous study that reported strong interest among Australian pharmacists in adopting stewardship roles in primary care.^33^

To our knowledge, this is the first study that evaluates the practices, facilitators, and barriers regarding AMS in community pharmacies in south-south Nigeria. Consequently, the findings offer locally relevant and actionable insights into AMS at the community level. However, several limitations should be noted. The cross-sectional design may limit causal inferences, and reliance on self-reported behaviors may be subject to social desirability bias. Furthermore, as the study was conducted in only one state, this may limit the generalizability of the findings.
